# Diversity and Variation of Bacterial Community Revealed by MiSeq Sequencing in Chinese Dark Teas

**DOI:** 10.1371/journal.pone.0162719

**Published:** 2016-09-30

**Authors:** Jianyu Fu, Haipeng Lv, Feng Chen

**Affiliations:** 1 Key Laboratory of Tea Plants Biology and Resources Utilization of Agriculture Ministry, Tea Research Institute, Chinese Academy of Agricultural Sciences, Hangzhou, 310008, PR China; 2 Graduate School of Chinese Academy of Agricultural Sciences, Beijing, 100081, PR China; 3 Department of Plant Sciences, University of Tennessee, Knoxville, TN, 37996–4561, United States of America; University of Illinois at Urbana-Champaign, UNITED STATES

## Abstract

Chinese dark teas (CDTs) are now among the popular tea beverages worldwide due to their unique health benefits. Because the production of CDTs involves fermentation that is characterized by the effect of microbes, microorganisms are believed to play critical roles in the determination of the chemical characteristics of CDTs. Some dominant fungi have been identified from CDTs. In contrast, little, if anything, is known about the composition of bacterial community in CDTs. This study was set to investigate the diversity and variation of bacterial community in four major types of CDTs from China. First, the composition of the bacterial community of CDTs was determined using MiSeq sequencing. From the four typical CDTs, a total of 238 genera that belong to 128 families of bacteria were detected, including most of the families of beneficial bacteria known to be associated with fermented food. While different types of CDTs had generally distinct bacterial structures, the two types of brick teas produced from adjacent regions displayed strong similarity in bacterial composition, suggesting that the producing environment and processing condition perhaps together influence bacterial succession in CDTs. The global characterization of bacterial communities in CDTs is an essential first step for us to understand their function in fermentation and their potential impact on human health. Such knowledge will be important guidance for improving the production of CDTs with higher quality and elevated health benefits.

## Introduction

Tea, produced from tea plant *Camellia sinensis* (L.) O. Kuntze, is one of the most popular beverages worldwide. First used by Chinese as medicine almost 5000 years ago, nowadays tea is being consumed for many health benefits, such as weight loss promotion, chronic illnesses prevention and even mood improvement [1–3]. In general, Chinese teas are divided into six categories according to the processing technology: a) green tea (no oxidation); b) white tea (slightly oxidized; c) yellow tea (lightly oxidized; d) oolong tea (partially oxidized); e) black tea (fully oxidized); f) dark tea (post-fermented) [4]. These six types of teas have different health benefits because of their characteristic chemical components.

Chinese dark teas (CDTs) are usually produced in the southwest areas of China and traditionally consumed mostly by ethnic minorities around border regions hundreds of years ago. Now they are popular in China and even being accepted by more people of other countries because of their health benefits [5]. Basically, the unique ‘piling store’ technology is the post-fermentation process that is a critical step for the production of CDTs [6]. In this process, certain microorganisms enter into tea leaf materials and trigger the fermentation that is considered being important to the chemical quality of CDTs [5–8]. Compared to other teas, some chemical components such as catechins and L-theanine in CDTs declined dramatically, and the representative catechins oxidation products theaflavins and thearubigins were undetectable in CDTs [6]. The unique chemistry of CDTs has been suggested to determine their distinct health-promoting effect, such as modulating intestine bacteria and improving the digestive system [9–11]. During the past decades, the microorganisms involved in post-fermentation process of dark teas and their roles for chemical characteristics have caused much attention [7, 12, 13]. Fuzhuan brick tea and Pu’er tea are two types of CDTs that have been studied for their associated microorganisms [12, 14, 15]. A dozen fungi, including *Aspergillus*, *Penicillium* and *Eurotium*, had been identified from CDTs using culture-dependent method or denaturing gradient gel electrophoresis (DGGE) technology [5, 8]. Some of these microorganisms are known to secrete some enzymes such as hydrolases, which may promote tea fermentation [16, 17]. In contrast to the knowledge about the fungal community of CDTs, little, if anything, is known about the composition of bacterial community in CDTs. Because many empirical fermented food and beverages including Kombucha tea are known to contain a rich bacterial community [18–23], we hypothesize that a rich bacterial community is associated with CDTs.

The previous studies on microbes of CDTs most focus on fungi in Fuzhuan brick-tea and Pu’er tea [5, 14, 24], this present investigation included another two major CDTs of Qingzhuan brick-tea and Liubao tea. Furthermore, the frequently used PCR-DGGE method in CDTs had its limitation to uncover the complete microorganism community [15, 24]. Recently, more and more microbial communities have been investigated by high-throughput sequencing as MiSeq in organisms, environment and foods, which enabled us to detect massive microbial populations at a high throughput and low cost way [20, 25, 26]. Instead of conventional methods based on physiological and morphological characteristics, this study employed MiSeq sequencing to identify the CDT-associated bacterial strains in four major types of CDTs. This comparative analysis enabled us to understand whether the specific environment in different geographical locations plays a critical role in the composition of the bacterial community associated with CDTs.

## Materials and Methods

### Ethics Statement

No specific permits were required for this study. As the sampling locations were not privately owned or protected in any way, and Chinese dark teas (CDTs) samples used in this study were traditional beverage and bought from tea shops or factories.

### CDTs samples collection

All tea samples were collected from major CDT-producing regions in China. Fuzhuan brick-tea samples were collected from Hunan Province and Zhejiang Province. Qingzhuan brick-tea samples were collected from Hubei Province. Pu’er tea samples were collected from Yunnan Province. Liubao tea samples were collected from Guangxi province S1 Table. The raw materials of CDTs were traditionally from two major varieties of tea plant: *C*. *sinensis* var. *sinensis* was used for processing Fuzhuan brick-tea, Qingzhuan brick-tea and Liubao tea teas, and *C*. *sinensis* var. *assamica* was used for processing Pu’er tea [6]. All the dark tea samples were commercial products and they were processed following traditional technologies.

### DNA extraction and PCR amplification

Microbial DNAs were extracted from one gram of tea material of each sample using the E.Z.N.A.^®^ Soil DNA Kit (Omega Bio-tek, Norcross, GA, U.S.) according to manufacturer’s protocols. The primers 338F: 5’-barcode-ACTCCTACGGGAGGCAGCAG-3’ and 806R: 5’-barcode-GGACTACHVGGGTWTCTAAT-3’ were used to amplify bacteria V3+V4 region of 16S ribosomal RNA gene [27], and the barcode was an eight-base sequence unique to each sample. The reaction condition was 95°C for 3 min, followed by 35 cycles: 95°C for 30 sec, 55°C for 30 sec, then extension at 72°C for 5 min. All PCR reactions were performed in triplicate in 20 μL mixture containing 4 μL of 5 × FastPfu Buffer, 2 μL of 2.5 mM dNTPs, 0.8 μL of each primer (5 μM), 0.4 μL of FastPfu Polymerase, and 10 ng of template DNA.

### Sequences preparation and Illumina MiSeq sequencing

The PCR products were extracted from 2% agarose gels and purified using the AxyPrep DNA Gel Extraction Kit (Axygen Biosciences, Union City, CA, U.S.) according to the protocol and quantified using QuantiFluor^™^-ST (Promega, U.S.). The purified amplicons were then pooled in equimolar and Illumina adapters were added by ligation (TruSeq DNA LT Sample Prep Kit); the products were further amplified with 10 cycles to obtain a sufficient yield for sequencing. The sequences were finally paired-end sequenced (2 × 250) on an Illumina MiSeq platform according to the standard protocols. The raw reads were deposited into the NCBI Sequence Read Archive (SRA) database (Accession Number: SRP080025).

### Processing of sequencing data

The raw fastq files were demultiplexed and quality-filtered using QIIME (version 1.17) with the following criteria: a) The 250 bp reads were truncated at any site receiving an average quality score less than 20 over a 10 bp sliding window, discarding the truncated reads that were shorter than 50bp; b) Exact barcode matching, two nucleotide mismatch in primer matching, reads containing ambiguous characters were removed; c) Only sequences that overlap longer than 10 bp were assembled according to their overlap region. Reads that could not be assembled were also discarded.

Operational Taxonomic Units were clustered with 97% similarity cutoff using UPARSE (version 7.1 http://drive5.com/uparse/) and chimeric sequences were identified and removed using UCHIME. The phylogenetic affiliation of each 16S rRNA gene sequence was analyzed by RDP Classifier (http://rdp.cme.msu.edu/) against the silva (SSU115) 16S rRNA database using confidence threshold of 70% [28].

## Results

### Sequence analysis by MiSeq sequencing

Four major types of CDTs from China: Fuzhuan brick-tea (FZ), Qingzhuan brick-tea (QZ), Pu’er tea (PR) and Liubao tea (LB), were selected for this study S1 Fig. A total of eleven CDT samples representing these four types of CDTs were collected from different geographyical regions. Four samples of FZ, two samples of QZ, three samples of PR and two samples of LB were subject to genomic DNA extraction and MiSeq sequencing. Totally, 377768 clean tags of 16S rRNA V4-V5 region were obtained from the 11 samples by MiSeq sequencing. 1457 Operational Taxonomic Units (OTUs) were generated after clustering at a 97% similarity level (Table 1). MiSeq sequencing had a high level coverage of over 99.9% among all CDTs samples. The bacterial sequences identified from CDTs were classified into phylum, family and genus levels. The bacterial communities from FZ and QZ were most diverse with each type of CDTs containing 12 phyla of bacteria. In contrast, at both the family and genus levels, the bacterial community from PR was the most diverse among the four types of CDTs containing 174 genera of bacteria that belong to 98 families. LB tea exhibited the least diversity in bacterial community containing 7 phyla, 36 families and 46 genera of bacteria S2 Table.

**Table 1:** Numbers of sequences analyzed, Operational Taxonomic Units (OTUs), estimated OUT richness (Chao), sample coverage, and diversity indices of Shannon and Simpson were calculated for 16S rRNA of 11 CDTs samples.

Sample ID	Reads	OTUs	Ace	Chao	Coverage	Shannon	Simpson
FZ1	34089	106	124	120	0.9993	2.23	0.2055
FZ2	30987	113	130	129	0.9993	1.76	0.3844
FZ3	31922	122	134	134	0.9994	1.92	0.3511
FZ4	39741	131	139	138	0.9996	2.31	0.2238
LB1	32546	42	68	55	0.9996	0.66	0.6592
LB2	38695	67	155	103	0.9993	0.71	0.6580
PR1	31679	249	261	264	0.9992	3.42	0.0922
PR2	32997	151	170	174	0.9993	2.8	0.1199
PR3	31028	185	205	214	0.9991	3.12	0.0882
QZ1	34998	148	157	159	0.9995	1.74	0.4084
QZ2	39086	143	152	151	0.9996	2.04	0.3350

FZ1-4, four samples of Fuzhuan brick tea; QZ1-2, two samples of Qingzhuan brick tea; PR1-3, three samples of Pu’er tea; LB1-2, two samples of Liubao tea.

**Fig 1:**
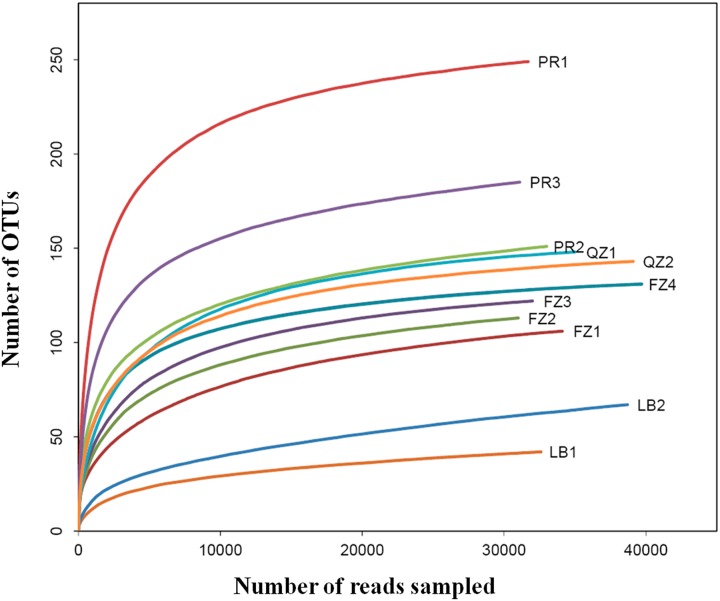
Rarefaction analyses for the observed number of OTUs from 11 dark tea samples at a genetic distance of 3%. The rarefaction curves for each sample of CDTs were displayed by different colors. FZ1-4, four samples of Fuzhuan brick tea; QZ1-2, two samples of Qingzhuan brick tea; PR1-3, three samples of Pu’er tea; LB1-2, two samples of Liubao tea.

The rarefaction analysis was performed to evaluate whether OTUs had been sufficiently recovered by MiSeq sequencing. Individual rarefaction curves corresponding to individual tea samples displayed a similar pattern of reaching a saturation phase [20]. At the numbers of reads generated for each sample, the numbers of OUTs were in the saturation phase (Fig 1), indicating that OUTs from each sample had been sufficiently recovered in MiSeq sequencing.

### Diversity of bacterial communities in CDTs

The richness of bacterial community for each sample was calculated by Ace and Chao estimators, and the community diversity was shown by Shannon and Simpson indices (Table 1). Samples within the same category shared much more similar bacterial richness and diversity than with the samples from other categories. For instance, the values of reads and OTUs, Ace and Chao estimators, and Shannon and Simpson indices of QZ1 were comparable to those of QZ2. Although FZ4 produced the most reads at 39741, the most number of OTUs (249) among all samples were identified from PR1. The other two samples of Pu’er tea, PR2 and PR3, also produced more OTUs than other samples (Table 1). Therefore, Pu’er tea possessed the highest bacterial richness and diversity among all the categories of CDTs. Moreover, LB1 and LB2 contained the least OTUs despite large numbers of reads, indicating that Liubao tea had the least bacterial diversity.

Rarefaction was also a suitable tool for microbial diversity studies [29]. The rarefaction curves calculated at 97% levels showed that the order of OTUs numbers from high to low among CDTs was PR > QZ > FZ > LB (Fig 1). The microbial richness based on rarefaction curves was also strongly supported by statistical diversity estimates, as the Ace, Chao, Shannon and Simpson values of three PR tea samples were 170–261, 174–264, 2.8–3.42, and 0.0882–0.1199, respectively (Table 1).

### Composition of bacterial communities in CDTs

**Fig 2:**
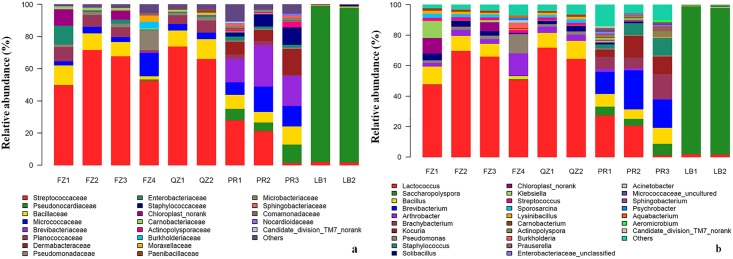
The bacterial communities of CDTs at family level (a) and genus level (b). Bacterial families were indicated by different colors, and “others” presented the rest families with a relative abundance less than 1%. FZ1-4, four samples of Fuzhuan brick tea; QZ1-2, two samples of Qingzhuan brick tea; PR1-3, three samples of Pu’er tea; LB1-2, two samples of Liubao tea.

The bacterial communities from the 11 samples of CDTs were analyzed at phylum, family and genus levels. In total, all the bacteria identified were classified into 238 genera, 128 families and 14 phyla. FZ and QZ teas shared similar bacterial communities, and they appeared to be significantly different with those of PR and LB teas. Most samples from the same type of CDTs shared high similar bacterial communities at all classification levels (Fig 2a and 2b).

*Firmicutes* and *Actinobacteria* were generally dominant bacterial phyla. They however had different distributions in different types of CDTs. *Firmicutes* was the dominant phylum in both FZ and QZ teas, and its proportions in the two teas were from 59% to 92%. *Actinobacteria* was the extremely dominant bacteria in Liubao tea, and its proportions in two samples were 96% and 97%. In addition, *Firmicutes* and *Actinobacteria* had roughly equal distributions in Pu’er tea.

Amongst 128 families, 21 of them had a relative abundance of larger than 1% in CDTs. The taxon result showed that *Streptococcaceae* was the dominant family in FZ and QZ teas with 50%-74% abundance, and *Pseudonocardiaceae* was the extremely dominant family in LB tea with 96% and 97% abundance. In addition to *Streptococcaceae* and *Pseudonocardiaceae*, PR tea contained seven relatively dominant bacteria populations and their average proportions in the three samples of PR were about 10%. At genus level, *Lactococcus* was the dominant bacteria in FZ and QZ teas, and *Saccharopolyspora* was the extremely dominant genus in LB tea. Pu’er tea samples also contained seven dominant genera that were *Lactococcus*, *Saccharopolyspora*, *Bacillus*, *Brevibacterium*, *Brachybacterium*, *Kocuria* and *Staphylococcus* (Fig 2b). Thus, Pu’er tea had a more diverse bacterial community than other dark teas at the phylum, family and genus levels.

### Phylogenetic analysis of bacterial communities in CDTs

Hierarchically heat maps for bacterial communities based on Bray-Curtis distance at phylum, family and genus levels were constructed. From the heat map at the genus level, FZ and QZ teas showed similar bacterial structures, which were different with that of LB or PR tea. Evidently, the heat map indicated that the samples from the same type of CDTs showed highly similar bacterial composition at genus level.

**Fig 3:**
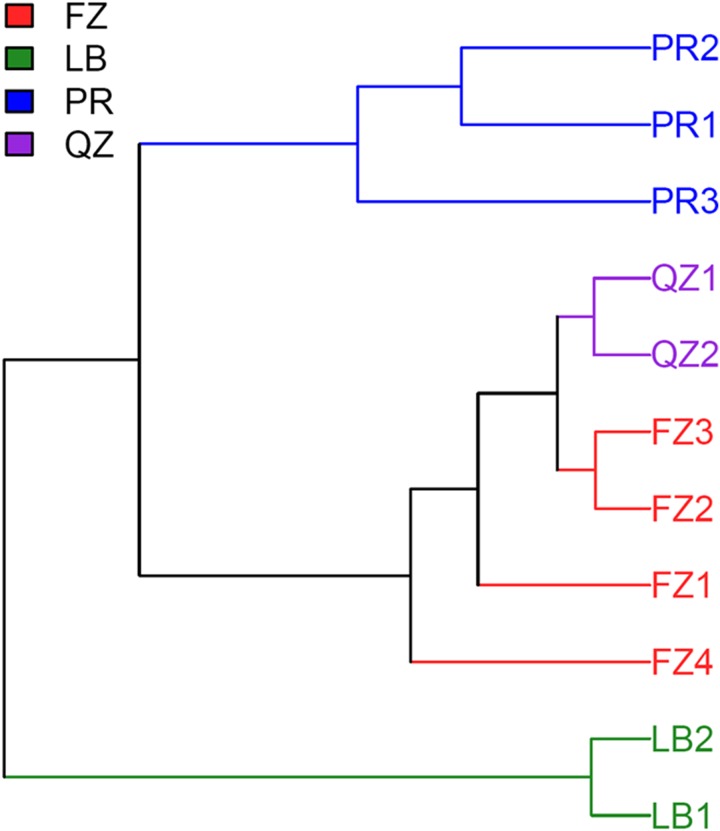
The hierarchical cluster tree based on Bray-Curtis distance for bacterial OTUs from CDTs. PR, QZ, FZ and LB tea samples were indicated by blue, purple, red and green colors, respectively. FZ, Fuzhuan brick tea; QZ, Qingzhuan brick tea; PR, Pu’er tea; LB, Liubao tea.

**Fig 4:**
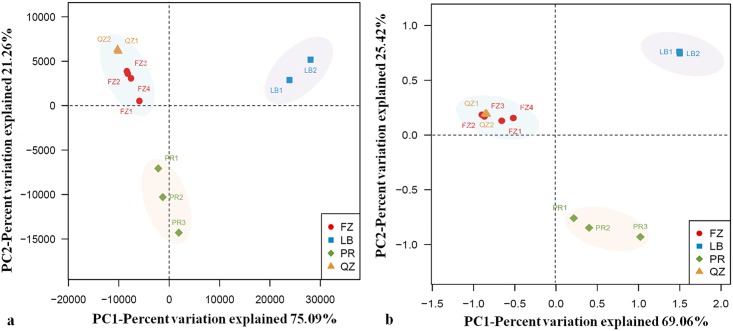
The plots of principal component analysis (PCA) (a) and principal coordinate analysis (PCoA) based on unweighted UniFrac distance metric (b). Samples of CDTs were presented by different color-filled symbols. FZ1-4, four samples of Fuzhuan brick tea; QZ1-2, two samples of Qingzhuan brick tea; PR1-3, three samples of Pu’er tea; LB1-2, two samples of Liubao tea.

Next, the taxonomic composition of bacterial communities for CDTs based on OTUs was analyzed. Hierarchical cluster tree showed that the bacteria in PR and LB teas were two separate clades, while that of FZ and QZ teas were in the same clade (Fig 3). The bacterial communities of different CDTs were also compared using multivariate principal component analysis (PCA). The four types of CDTs formed three clusters: one cluster contained FZ and QZ teas; the other two separate clusters were constituted by PR and LB teas, respectively (Fig 4a). Similarly, the bacterial communities at the OTU-level of CDTs were compared using principal coordinate analysis (PCoA) based on the unweighted and weighted UniFrac distances. The plot disclosed that the communities of two brick teas clustered together, but that of PR and LB teas were different significantly and as separate clusters (Fig 4b).

The overlaps of OTUs clusters among four categories of CDTs were calculated after singleton sequences being removed. The Venn diagrams revealed consistent overlap patterns between each two types of CDTs, and it showed all CDTs shared 53 OTUs that was about 22% of total bacterial genera identified. FZ, QZ and PR teas shared a high degree overlap with each other that were from 126 to 130 OTUs, while each of them had a lower overlap with LB tea from 61 to 68. It suggested that the bacterial community in LB tea was much distinct with the other CDTs categories.

## Discussion

Diverse and rich communities of bacteria and fungi with potential health benefits have been identified from various fermented food and beverages including tea [19, 23]. Microorganisms in CDTs are critical for the distinctive biochemical characteristics of dark teas. The increasing popularity and market demand of dark teas requires a better understanding of the roles of microorganisms in CDTs production, the tea quality and the health promoting effect of dark teas. While previous studies of the microbial community of CDTs had focused on fungi [14], the present study aimed at identifying the bacteria communities associated with CDTs.

As for the bacteria communities, an earlier dynamic study identified ten bacteria families belong to four phyla in PR tea using PCR-DGGE method [15]. Here, the analysis of 11 samples from four types of CDTs using MiSeq sequencing revealed an extensively diverse bacterial community in CDTs, which included bacteria from 238 genera, 128 families and 14 phyla. Compared to the approximately 20 genera of fungi that have been identified from CDTs [5, 6, 12, 24], the bacterial community associated with CDTs seems to be much more diverse.

Our comparative analysis (Fig 4) showed that FZ and QZ teas, two types of brick teas from adjacent regions, shared similar bacterial compositions; whereas PR and LB teas had distinct bacterial composition profiles, which were also significantly different with that of FZ and QZ teas. It had been known that microorganisms in CDTs were usually determined by the processing and the storage environment [6], which had been proved in previous fungal studies [5, 12]. In the present study, the samples within LB, QZ or PR were found to share nearly identical bacterial communities (Fig 2), because they were processed in adjacent regions with approximate tea making conditions and environments. Even the samples from FZ and QZ teas still shared high similar bacterial compositions, which might be due to their closer processing sites with a similar microbiological flora. Thus, the producing area environment was one of the major factors that determine the bacteria populations in CDTs. Interestingly, FZ4 from a relatively isolated site also had a similar bacterial structure as other three FZ samples. This suggests that the processing technologies might have an effect on microorganism population and adaptation in CDTs. *C*. *sinensis* var. *sinensis* is the most widely planted variety in China and it was traditionally used for processing FZ, QZ and LB teas. *C*. *sinensis* var. *assamica* is mainly grown around Yunnan province, and it has been used for processing Pu’er tea. Nevertheless, tea blending has often been employed during dark tea processing. Therefore, it remains to be determined whether tea material has a major effect on the microbial community associated with CDTs.

With the identification of a rich and diverse community of bacteria associated with CDTs, it is a legitimate question to ask what the impact of such bacterial community on health benefits as well as on tea safety. To date, 18 beneficial bacterial families containing 28 genera have been detected from fermented food and beverages [19]. Except the family of *Acetobacteraceae*, all the other 17 beneficial families covering 20 genera on the list were detected from CDTs. Furthermore, these beneficial bacteria had an overwhelming numerical superiority in relevant bacterial communities. *Lactococcus* has been well known for milk fermentation, it probably converts some chemical components and synthesized aroma substances, and it also has antifungal activity that could resist some harmful fungi to keep a stable microorganism succession [18, 30]. It also has an important presence in the bacterial community of FZ and QZ teas (Fig 2). Therefore, *Lactococcus* might have a positive effect on post-fermentation in CDTs. *Bacillus* also constituted a large portion in bacterial communities of CDTs that was similar as fermented coffee, and it could produce a series of extracellular enzymes that contribute to depolymerizing the cellulose-containing complexes and pectin during post-fermentation in CDTs [22, 31]. In summary, it was clear that the predominant bacteria in CDTs were beneficial strains and they might promote the fermentation reaction and have health benefits.

Fungi and bacteria comprise the major microbial community in dark teas, and each of them may play an irreplaceable role during dark tea fermentation. Their succession was mainly influenced by moisture, temperature, substrate competition, enzymatic capacity and antimicrobial activity during fermentation in CDTs [31]. The present study presents novel information about the bacterial community associated with CDTs using the MiSeq sequencing technology. In the future, great attention should be paid to the effects of such bacterial communities on dark tea fermentation and their impact on dark tea quality and human health.

## Supporting Information

S1 Fig The four typical Chinese dark teas (CDTs) used in this study. A: Fuzhuan brick tea, FZ; B: Qingzhuan brick tea, QZ; C: Pu’er tea, PR; D: Liubao tea, LB. The sample numbers of were four, two, three and two, respectively.
(TIF)

S2 Fig The venn diagram demonstrating OTUs cluster overlap among four types of CDTs. The OUTs with 97% smilarity between each two CDTs types were defined as shared OTUs. FZ, Fuzhuan brick tea; QZ, Qingzhuan brick tea; PR, Pu’er tea; LB, Liubao tea.
(TIF)

S1 Table The CDTs samples used in this study. FZ1-4, four samples of Fuzhuan brick tea; QZ1-2, two samples of Qingzhuan brick tea; PR1-3, three samples of Pu’er tea; LB1-2, two samples of Liubao tea.
(DOCX)

S2 Table The number of bacteria identified from four types of CDTs at phylum, family and genus levels. FZ, Fuzhuan brick tea; QZ, Qingzhuan brick tea; PR, Pu’er tea; LB, Liubao tea.
(DOCX)
