# Supplementary figures and images for: Diversity and Variation of Bacterial Community Revealed by MiSeq Sequencing in Chinese Dark Teas
Source: PLoS One. 2016 Sep 30;11(9):e0162719. doi: 10.1371/journal.pone.0162719 (PMC5045175; doi:10.1371/journal.pone.0162719)

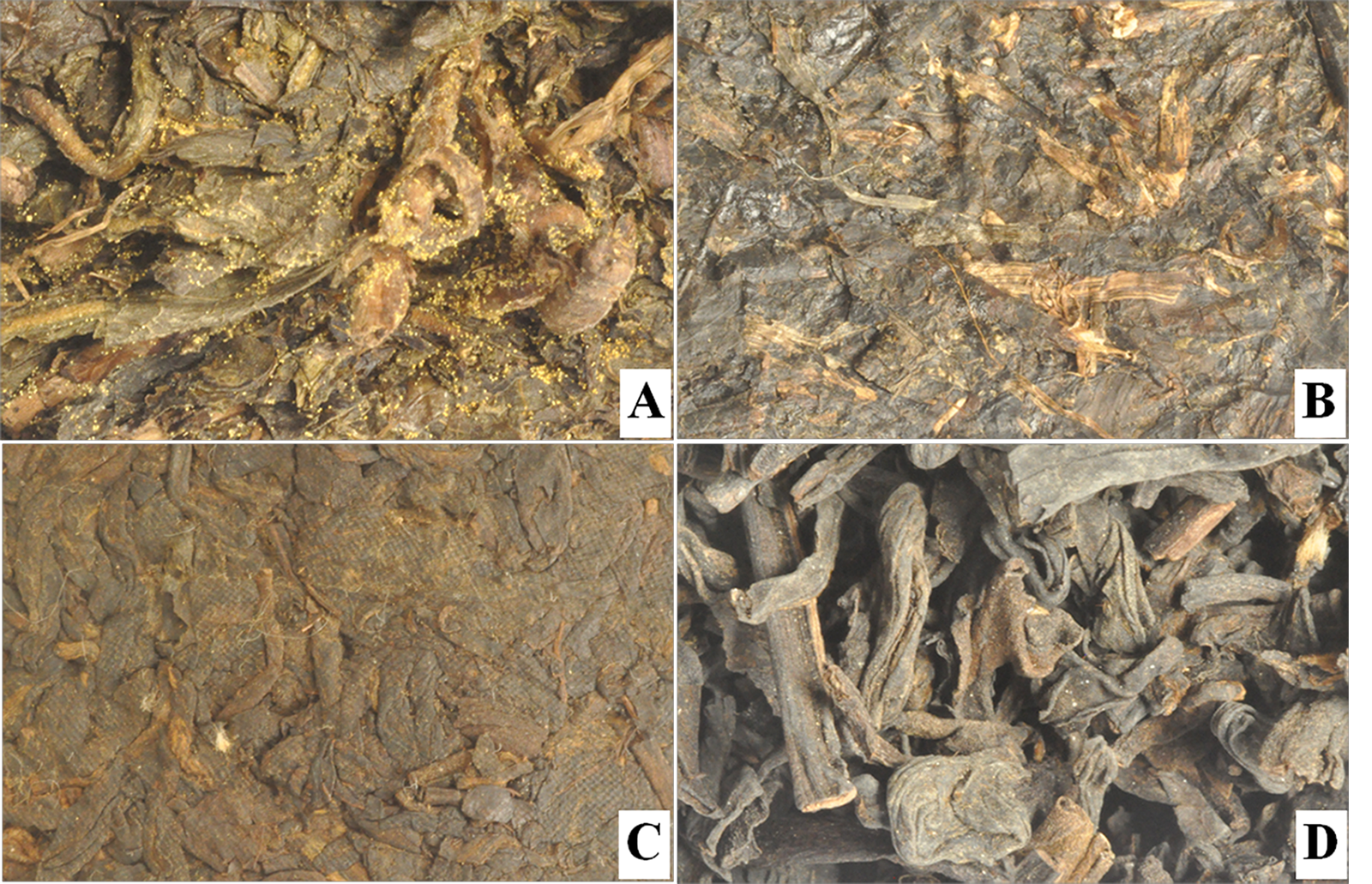

Supplement: S1 Fig — A: Fuzhuan brick tea, FZ; B: Qingzhuan brick tea, QZ; C: Pu’er tea, PR; D: Liubao tea, LB. The sample numbers of were four, two, three and two, respectively. (TIF) [file pone.0162719.s001.tif]

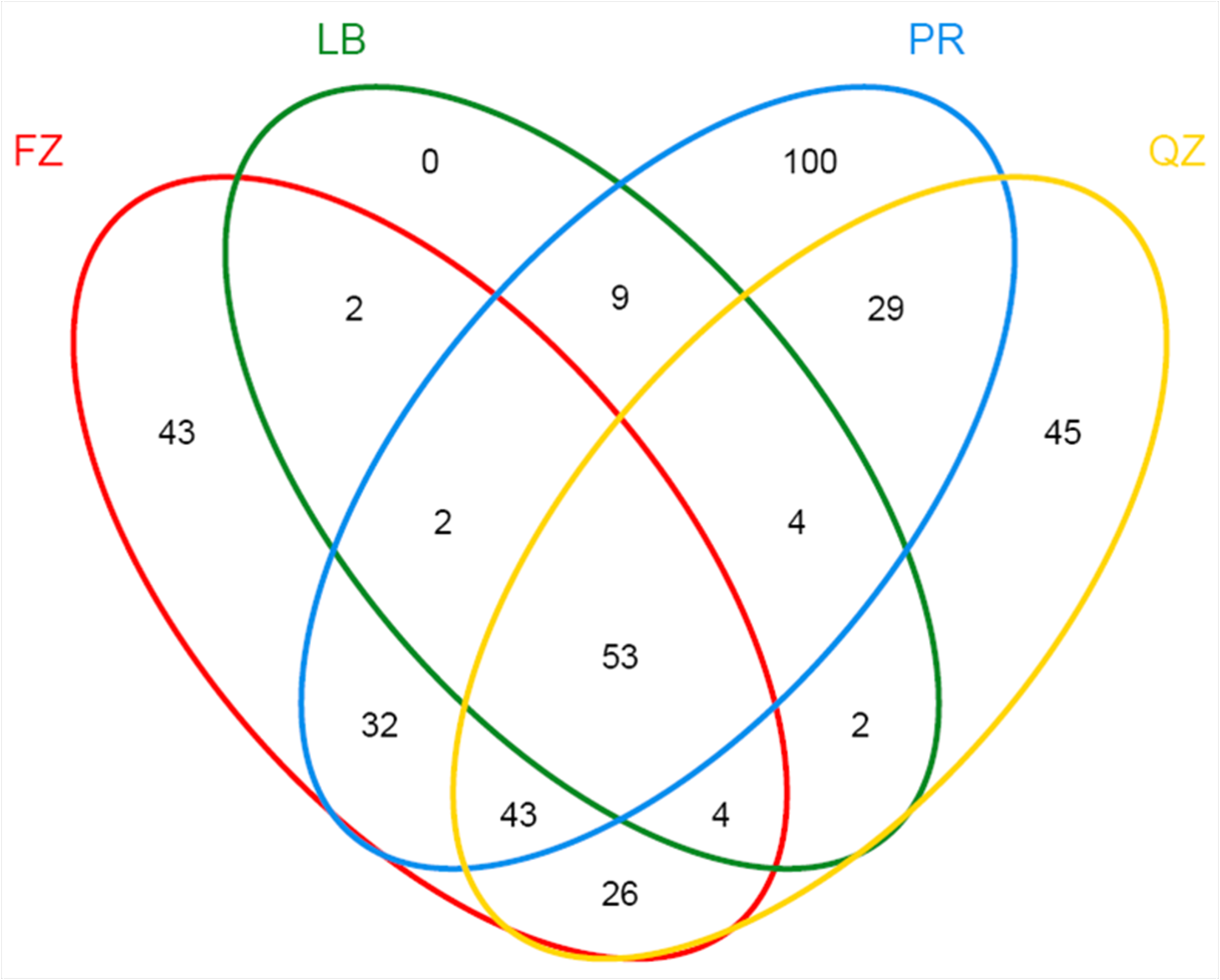

Supplement: S2 Fig — The OUTs with 97% smilarity between each two CDTs types were defined as shared OTUs. FZ, Fuzhuan brick tea; QZ, Qingzhuan brick tea; PR, Pu’er tea; LB, Liubao tea. (TIF) [file pone.0162719.s002.tif]
