# Supplementary material for: Diversity and Variation of Bacterial Community Revealed by MiSeq Sequencing in Chinese Dark Teas
Source: PLoS One. 2016 Sep 30;11(9):e0162719. doi: 10.1371/journal.pone.0162719 (PMC5045175; doi:10.1371/journal.pone.0162719)
Supplement: S1 Table — FZ1-4, four samples of Fuzhuan brick tea; QZ1-2, two samples of Qingzhuan brick tea; PR1-3, three samples of Pu’er tea; LB1-2, two samples of Liubao tea. (DOCX) [file pone.0162719.s003.docx]

**S1 Table. The CDTs samples used in this study.**

| **Sample ID** | **Category** | **Trademark** | **Factory** |
| --- | --- | --- | --- |
| LB1 | Liubao tea | Three Cranes | Wuzhou Tea Factory |
| LB2 | Liubao tea | Cang Wu | Cangwu Liubao Tea Industry Co. |
| PR1 | Pu’er tea | CNNP | Yunnan Division of China Tea Industry Co. |
| PR2 | Pu’er tea | Da Yi | Dayi Tea Factory |
| PR3 | Pu’er tea | CNNP | Kunming Tea Factory of China Tea Industry Co. |
| FZ1 | Fuzhuan brick tea | Xiang Yi | Yiyang Tea Factory |
| FZ2 | Fuzhuan brick tea | An Hua Hei Cha | Anhua Tea Factory |
| FZ3 | Fuzhuan brick tea | Bai Sha Xi | Baishaxi Tea Factory |
| QZ1 | Qingzhuan brick tea | Chuan Zi | Zhaoliqiao Tea Factory |
| QZ2 | Qingzhuan brick tea | Dong Zhuang | Chibi Dongzhuang Tea Industry Co. |

FZ1-4, four samples of Fuzhuan brick tea; QZ1-2, two samples of Qingzhuan brick tea; PR1-3, three samples of Pu’er tea; LB1-2, two samples of Liubao tea.
