# Supplementary material for: Diversity and Variation of Bacterial Community Revealed by MiSeq Sequencing in Chinese Dark Teas
Source: PLoS One. 2016 Sep 30;11(9):e0162719. doi: 10.1371/journal.pone.0162719 (PMC5045175; doi:10.1371/journal.pone.0162719)
Supplement: S2 Table — FZ, Fuzhuan brick tea; QZ, Qingzhuan brick tea; PR, Pu’er tea; LB, Liubao tea. (DOCX) [file pone.0162719.s004.docx]

**S2 Table. The number of bacteria identified from four types of CDTs at phylum, family and genus levels.**

| CDTs type | Phylum | Family | Genus |
| --- | --- | --- | --- |
| FZ | 12 | 78 | 130 |
| QZ | 12 | 79 | 128 |
| PR | 10 | 98 | 174 |
| LB | 7 | 36 | 46 |

FZ, Fuzhuan brick tea; QZ, Qingzhuan brick tea; PR, Pu’er tea; LB, Liubao tea.
